# Oral health among children attending an oncology clinic in Trinidad

**DOI:** 10.1002/cre2.232

**Published:** 2019-08-16

**Authors:** Anne Kowlessar, Rahul Naidu, Visha Ramroop, Janelle Nurse, Kavita Dookie, Curt Bodkyn, Sanjay Lalchandani

**Affiliations:** ^1^ School of Dentistry University of the West Indies, St. Augustine Campus St. Augustine Trinidad and Tobago; ^2^ Department of Medical Sciences Eric Williams Medical Sciences Complex Mt Hope Trinidad and Tobago

**Keywords:** neoplasm, oral health, paediatrics

## Abstract

**Objectives:**

Little is known about the oral health of paediatric oncology patients in the Caribbean. Poor oral health can complicate oncology treatment, negatively affecting a child's health. In children undergoing chemotherapy or bone marrow transplant, odontogenic infections can progress to life‐threatening sepsis. The aim of this study is to investigate the oral health among children attending an oncology clinic in Trinidad.

**Material and Methods:**

Sample population included paediatric oncology patients attending a children's hospital in Trinidad. Subsequent to obtaining informed consent, a 14‐item questionnaire was administered to parents/caregivers. An intra‐oral examination was undertaken by two dentists to assess soft tissues, gingival health, and dentition status, using visual examination only.

**Results:**

Seventy‐one children and their caregivers participated in the study. The children consisted of both patients warded or attending as outpatients; 53.5% of patients were male and the mean age 6.64 (SD 3.33) years with a range of 1 to 15 years. Gingivitis and mucositis were present among 41.3% and 3% of patients, respectively. The prevalence of visible dental caries was 54.3%. Caries experience (dmft) was 2.28 (SD 3.63), and for those children with some caries experience (dmft > 0), this was 5.59 (SD 3.72). The majority (62.5%) had never visited a dentist. The most common dental treatment needs were dental prophylaxis (98.4%) and restorative treatment (50.8%). Acute lymphocytic leukaemia (39.1%) was the most common malignancy among this sample, and patients were at varying stages of cancer treatment.

**Conclusions:**

Oral health among this sample of paediatric oncology patients was generally poor, with untreated caries being common, and the majority of children not having had any previous dental care. Preventive dental care for these patients should include oral hygiene instruction, dietary advice, topical fluoride application along with management of carious lesions, and odontogenic infections. This preliminary study highlights the need for closer collaboration between general dental practitioners, paediatric dentists, and paediatric oncology physicians, in caring for these patients.

**Why is this paper important to Paediatric Dentists?**

This paper is important as it shows the need for collaboration between paediatric oncologists and paediatric dentists, which is essential to maintain optimal oral health among paediatric oncology patients and to minimize oral complications arising from cancer and its treatment.

## 1 INTRODUCTION

The International Agency for Research on Cancer indicates that approximately 300,000 cases of cancer are diagnosed in children and teens under the age of 19 every year with an estimated 80,000 deaths occurring annually from childhood cancer worldwide making cancer the leading cause of disease‐related fatalities for children between 1 and 14 years. (International Agency for Research on Cancer, n.d.)

Global cancer estimates of cancer incidence and mortality suggest that there will be an estimated 18.1 million new cancer cases (17.0 million excluding non‐melanoma skin cancer) and 9.6 million cancer deaths (9.5 million excluding non‐melanoma skin cancer) in 2018. (Bray et al., 2018)

Chemotherapy and/or radiotherapy for the treatment of cancer may cause many acute and long‐term side effects in the oral cavity. Furthermore, because of the immunosuppression that patients experience, any existing or potential sources of oral/dental infections and/or soft tissue trauma can compromise the medical treatment, leading to morbidity, mortality, and higher hospitalization costs. (American Academy of Pediatric Dentistry (AAPD), 2013) Prevention and treatment of preexisting or concomitant oral disease is essential to minimize complications in this population. (Elad, Thierer, Bitan, Shapira, & Meyerowitz, 2008)

Acute lymphocytic leukaemia is the most commonly occurring paediatric cancer. (Bray et al., 2018; American Academy of Pediatric Dentistry (AAPD), 2013) International studies have shown a high prevalence of dental caries among paediatric oncology patients, and the most common dental procedures amongst these patients included preventive and restorative procedures and removal of infectious foci. (Bray et al., 2018; Elad et al., 2008)

Poor oral health can complicate oncology treatment, negatively affecting a child's health. In children undergoing chemotherapy or bone marrow transplant, odontogenic infections can progress to life‐threatening sepsis.

Little is known about the oral health of paediatric oncology patients in the Caribbean. In Trinidad and Tobago, a twin island republic, located in the Caribbean, the crude incidence rate of childhood cancer for the period 2001–2006 was determined to be 1.9 per 100,000 patient years (pyrs). (Bodkyn & Lalchandani, 2010)

The aim of this study was to investigate the oral health among children attending an oncology clinic in Trinidad.

## 2 MATERIALS AND METHODS

The study involved a convenience sample of paediatric cancer patients attending the Paediatric Oncology service at the Eric Williams Medical Sciences Complex (EWMSC), which is the national treatment centre for all ages.

Ethical approval for the study was granted by the Ethics Committee of the University of the West Indies, and informed consent was obtained from parents/guardians of these oncology patients to be part of the study.

**Figure 1:**
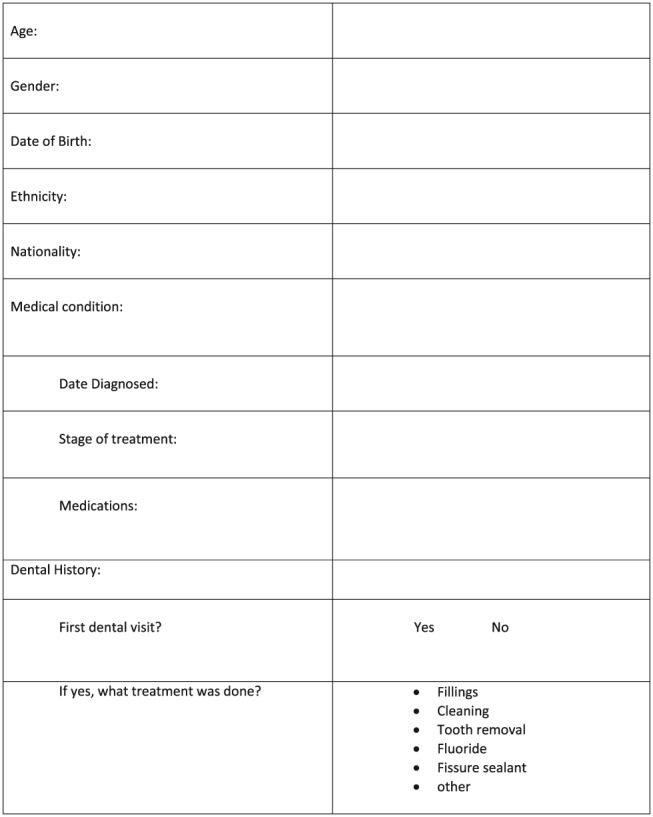
Questionnaire/data form

A 14‐item questionnaire (Figure 1) was used to record data on the age, sex, ethnicity, nationality, medical history, stage of treatment, medications taken, and previous dental treatment of these paediatric oncology patients.

A clinical examination was undertaken by a single calibrated examiner using a dental mirror and ambient light. Training and calibration of the examiner were undertaken in the paediatric dental clinic of the University of the West Indies with a specialist clinician as the gold standard.

Examination and criteria for caries diagnosis was based on WHO guidelines for oral health surveys. (WHO, 2013)

A brief extra‐oral examination was conducted assessing the facial tissues and nerves. An intra‐oral examination followed, investigating the oral soft tissues, in particular the gingiva and mucosa, for any signs of inflammation.

A simplified visual scoring for gingival health (presence/absence of inflammation) and mucositis was used due to some of the children being in‐patients on the oncology ward and having limited time for examination.

Dental charting included absence/presence of teeth, obvious carious teeth, and any filled teeth. Data were processed and analysed using SPSS (version 24).

Data collection occurred over the course of 3 years.

## 3 RESULTS

Seventy‐one children and their caregivers participated in the study. Patients were at varying stages of cancer treatment. The children consisted of both patients warded and those attending as outpatients children ranged in age from 1 to 15 years and the mean age was 6.64 (SD 3.33) years; 53.5% of patients were male. The main ethnic groups were African (52.4%), East Indian (34.9%), and mixed decent (12.7%).

Acute lymphocytic leukaemia (39.1%) was the most common malignancy among this sample. (Table 1).

**Table 1:** Frequency of medical conditions among participants (*N* = 71)

Medical diagnosis	Frequency	%	ICD‐10 code
Acute lymphocytic leukaemia	27	38.0	C91.00
Germ cell tumour	1	1.4	C62.90
Retinoblastoma	2	2.8	C69.21
B cell lymphoma	1	1.4	C85.12
Brain tumour	3	4.2	C71.90
Hodgkin's lymphoma	1	1.4	C81.90
Rhabdomyosarcoma	1	1.4	C49.90
Astrocytoma	2	2.8	C71.90
Wilms tumour	9	12.7	C64.90
Meduloblastoma	2	2.8	C71.90
Langerhans cell histocytosis	1	1.4	C96.60
Sarcoma	3	4.2	C49.90
Non‐Hodgkins lymphoma	2	2.8	C85.80
Glioneuroma	3	4.2	C71.90
Optic pathway glioma	1	1.4	C72.30
Intermediate pilomyxoid astrocytoma	2	2.8	C71.90
Hepatoblastoma	1	1.4	C22.20
Juvenile myelomonocytic leukaemia	1	1.4	C93.30
Ewings sarcoma	1	1.4	C41.90
Ganglioneuroblastoma	1	1.4	C49.90
Acute Myeloid leukaemia	2	2.8	C92.90
Burkett's lymphoma	1	1.4	C83.70
Neuroblastoma	1	1.4	C74.90

### 3.1 Oral health

The prevalence of visible caries was 54.3% in the primary dentition. In this dentition, mean caries experience (dmft) was 2.28 (SD 3.63), and for those children with some caries experience (dmft > 0), this was 5.59 (SD 3.72). Caries experience came entirely from decayed teeth (dt), ranging from 1 to 20, with 25 children having three or more decayed teeth. Seven children between the ages of 6 and 13 years old presented with caries in their permanent dentition; 54% of children had first permanent molars.

Table 2 describes caries experience for the primary and permanent dentition (dmft/DMFT) by age group, with highest severity in the 6‐ to 8‐year‐olds.

**Table 2:** Caries experience (dmft/DMFT) by age group (N = 71)

Age group (years)	*n*	dmft (SD)	DMFT (SD)
1–5	26	1.65 (2.95)	—
6–8	24	3.5 (4.63)	0.08 (0.41)
9–11	12	2.67 (3.31)	0.17 (0.39)
12–15	9	—	0.22 (0.67)

For the whole sample, the proportion of children with gingivitis and mucositis was 41.3% and 3%, respectively.

The majority (62.5%) of patients had never visited a dentist. The most common dental treatment needs were dental prophylaxis (98.4%) and restorative treatment (50.8%).

## 4 DISCUSSION

The findings of this study are consistent with international data that shows children with cancer have a high prevalence of oral diseases including caries, gingivitis, and mucositis. (Al‐Dakkak, 2011; Sonis & Fey, 2002; Velten, Zandonade, & de Barros Miotto, 2017) The present study also suggests that paediatric oncology patients are at increased risk of caries as over half had visible caries, with a third having three or more decayed teeth. The prevalence of caries experience in this sample was lower than that reported in the last national survey of schoolchildren for those aged 6–8 years (Naidu, Prevatt, & Simeon, 2006); however, the small sample and wider age range in the present study limit direct comparison.

It should be noted that the prevalence of gingivitis in the sample may have been underestimated due to the examinations taking place in nonideal conditions. Due also to the nondental setting, it was felt that plaque assessment would not be reliable.

Cancer and its associated treatments reduce a child's ability to fight infections, and similarly, any existing or potential sources of oral/dental infections and/or soft tissue trauma can compromise the medical treatment, which may lead to morbidity, mortality, and higher hospitalization costs. In addition, chemotherapy and/or radiotherapy for the treatment of cancer may cause many acute and long‐term side effects in the oral cavity. (National Cancer Institute, n.d.) As a result, the high levels of untreated dental decay reported for children is our study have implications for the successful administration of cancer treatment and consequent quality of life among these patients. Early referral and evaluation of patients should allow for the early detection of any dental disease and possibly less invasive treatments options with an aim to reduce the complications of dental treatment. In addition, early evaluation of these patients would provide the opportunity to implement appropriate preventive dental advice and treatment, which could reduce the development of future dental disease.

Acute side effects and systemic complications of cancer treatment may include pain, mucositis, oral ulcerations, bleeding, taste dysfunction, secondary infections (e.g., candidiasis and herpes simplex virus), salivary gland dysfunction (e.g., xerostomia), neurotoxicity and mucosal fibrosis, post‐radiation osteonecrosis, soft tissue necrosis, temporomandibular dysfunction (e.g., trismus), and craniofacial and dental developmental anomalies (American Academy of Pediatric Dentistry, 2013). This again highlights the need for close dental evaluation of these patients so that quality of life can be preserved as much as possible.

Research has also shown that the effects of cancer and its treatment can manifest years later in the form of developmental dental disturbances such as enamel mineralization disturbances and root malformation (Avşar, Elli, Darka, & Pinarli, 2007) and microdontia, hypodontia, and taurodontia (Vaughan et al., 2005). The frequency of these factors was determined in relation to age at initiation of treatment, (Kaste et al., 1997) and the severity of the long‐term effects is dependent on the age of the child at initiation of treatment and whether chemotherapy is combined with radiation or not (Raja Zarina & Nik‐Hussein, 2005). These long‐term effects suggest that paediatric cancer patients require monitoring not only during active disease but rather they should continue to be monitored closely even during remission in order to detect these potential problems and prevent complications such as difficulties with eating, speaking, aesthetics, and thus preserving the quality of life.

Therefore, the long‐term plan for these patients should include a carefully scheduled preventive programme that includes components of the four pillars of prevention such as dental prophylaxis, oral hygiene and dietary advice, fissure sealants, and the application of fluoride varnish at appropriate intervals.

Clinical guidelines produced by the Royal College of Surgeons of England (2018) recommend that all oncology protocol should include an early pretreatment oral assessment. The guidelines also recommend that during the acute phase of treatment, a trained dental professional charged with the responsibility of the patient's oral care must form part of the oncology team. Furthermore, the guidelines state that the oncology patient discharge protocol should include a procedure for ensuring continuing oral care.

These guidelines emphasize the need for close collaboration between the oncology and dental team. This therefore highlights the need for a multidisciplinary approach in the management of the paediatric oncology patient. Dental care is important during all phases of cancer care and can be facilitated by appropriate referral pathways between the oncology department and dental team. This is particularly important in Trinidad given the high levels of untreated dental disease among paediatric oncology patients in this study, which highlights the need for the stream lining of the existing referral pathways and development of a protocol for the oral care for these patients.

## 5 CONCLUSION

Oral health among this sample of paediatric oncology patients was generally poor, with untreated caries being common and the majority of children not having had any previous dental care. Preventive dental care for these patients should include oral hygiene instruction, dietary advice, topical fluoride application along with management of carious lesions, and odontogenic infections. This study highlights the need for closer collaboration between paediatric dentists and paediatric oncology physicians, in caring for these patients.

## CONFLICT OF INTEREST

The authors have no conflict of interest to declare.

## AUTHOR CONTRIBUTIONS

All authors listed above have contributed to the design of the study. The authors would also like to acknowledge the University of the West Indies, St Augustine Campus Research and Publication Fund which provided a grant for this study. A. Kowlessar and J. Nurse collected the data. J. Nurse and K. Dookie analysed the data, and A. Kowlessar, R. Naidu, and V. Ramroop led the writing.
